# Magnetic Hyperbranched Molecular Materials for Treatment of Oily Sewage Containing Polymer in Oilfield Compound Flooding

**DOI:** 10.3389/fchem.2022.865832

**Published:** 2022-05-18

**Authors:** Sanyuan Qiao, Qingwang Liu, Zhenzhong Fan, Qilei Tong, Li Cai, Yuanfeng Fu

**Affiliations:** Petroleum Engineering, Northeast Petroleum University, Daqing, China

**Keywords:** sewage disposal, demulsification flocculation, magnetic separation, performance evaluation, magnetic hyperbranched material

## Abstract

With the continuous improvement in oilfield development and the application of tertiary oil recovery technology, the water content of oilfield-produced fluids has gradually increased, and a large number of oilfield sewage with complex components has also been produced after oil–water separation, and effective treatment is urgently needed. ASP flooding sewage contains alkali, various surfactants, polymers, microemulsion oil droplets, and solid impurities, which are difficult to be effectively treated by traditional water treatment agents and methods. In this study, aminopropyl triethoxysilane (APTES) was used to modify the nano-Fe_3_O_4_ coated with tetraethyl silicate (TEOS). The product was used as the ferromagnetic nano-core for the iterative reaction of Michael addition and ester amidation to synthesize a magnetic hyperbranched polyamide amine, and its performance in the treatment of ASP flooding wastewater was evaluated experimentally. For the preparation of APTES-modified Fe_3_O_4_@SiO_2_ (FOSN) product, TEOS was coated over Fe_3_O_4_ in an ethanol aqueous solution environment and then APTES was added dropwise. The first-generation branched product (1-FSMN) was obtained by the reaction of FOSN and methyl acrylate graft product (FOSN-M) with ethylenediamine, and the highest yield was 93.7%. The highest yield of the second-generation branched product (2-FSMN) was 91.6%. In this study, a composite flooding wastewater sample from a block in the Bohai oilfield was taken. The suspended solids content was 143 mg/L, the oil content was 921.09 mg/L, the turbidity was 135 NTU, and the zeta potential was −47 mV. The third-generation hyperbranched polymer (3-FSMN) and its quaternary ammonium salt (3-FSMN-Q) performed best in the appropriate dosage range, with the highest oil removal rate of 97%, suspended solid removal rate of 90.3%, turbidity reduction rate of 86.6% and zeta potential reduction rate of 88%. For 3-FSMN and its quaternary ammonium salt, the gravity/magnetic PAC compound treatment experiment was carried out. In the settlement time of only 5 min, 3-FSMN/PAC and 3-FSMN-Q/PAC can achieve the maximum oil removal rate of 87.1% and suspended solids removal rate of 87.3% for polymer containing wastewater from ASP flooding, and 86.3 and 86.0% for 120 mg/L. Its treatment capacity was much better than that of common treatment agent combination (CPAM/PAC).

## Introduction

The world’s energy consumption has grown significantly, and the demand for petroleum-based energy has also continued to grow. In order to improve crude oil recovery, the oilfield has implemented a ternary (polymer–surfactant–alkali) composite flooding technology on a large scale (Reddy, 2015). The ASP flooding oil recovery technology not only improves the crude oil recovery but also produces a large amount of composite flooding sewage (Wang et al., 2021). In addition to oil droplets, underground minerals, and solid impurities, the output sewage after oil–water separation also contains a certain amount of polymers, surfactants, and alkalis (Li et al., 2021). The synergistic action of oil droplets, polymers, surfactants, and alkalis in water will form stable emulsions, even microemulsions (Jin et al., 2021). The emulsion is stable in nature, difficult to break demulsification, slow to settle mechanical impurities in the water body, and difficult to flocculate (Chen et al., 2018; Huang et al., 2019). It is difficult for ordinary oilfield water treatment methods to separate oil droplets and suspended solids from stable emulsions or microemulsions.

The oil droplets of ASP flooding sewage have small average particle size, high viscosity, high degree of emulsification, and long time for oil–water separation in sewage (Yan et al., 2021). If the traditional natural sedimentation method or coagulation sedimentation method is still used for oil–water separation, the sedimentation time needs to be extended, but the excessively long residence time will make it difficult to control the volume of sedimentation equipment and occupy space resources (Zhu et al., 2018).

With the rapid development in nanomaterial research, the synthesis and application of nanomaterials have developed rapidly. Nanoparticles synthesized with magnetic iron oxide as the core have become popular magnetic nanomaterials, showing advantages in the research and application of different fields such as biochemistry (Ji et al., 2018; Feng et al., 2021). In order to prepare materials with certain physicochemical functions, the research on the synthesis, preparation methods, and physicochemical properties of nanoparticles with magnetic iron oxide as the core has also attracted extensive attention (Qi et al., 2021). Through strict quantitative control of experimental reaction conditions in synthesis, magnetic iron oxide nanoparticles with the following unique characteristics can be prepared (Wang et al., 2018; Duskey et al., 2021): dozens of nanometers in diameter, with special physical and biochemical properties, it can have superparamagnetism and no remanence, and it can be recycled and reused under the action of the magnetic field.

For the treatment of oilfield ASP flooding sewage, previous research has focused on biological treatment and conventional physical and chemical methods, such as gravity separation technology, hydrocyclone separation technology, coalescing air flotation technology, dosing air flotation technology, flocculant, and water quality modification (Boopathy and Raj, 2017; Zhang et al., 2017; Yang et al., 2021). The use of magnetic nanoparticles composite hyperbranched molecules to remove oilfield ternary composite flooding polymer-containing sewage is not yet mature. The main advantages of magnetic nanoparticles used in water purification are as follows: magnetic nanoparticles have high specific surface area and stable chemical properties and have strong adsorption performance for pollutants, and some adsorbents can be reused (Palchoudhury and Lead, 2014). The unique superparamagnetism realizes one-time adsorption and separation of pollutants in environmental water, avoiding the generation of secondary pollution. Hyperbranched polymer (HBP) has high application potential in many fields due to its unique physical and chemical properties brought by its highly branched structure (Sun et al., 2017). It also shows unique advantages in the purification and treatment of industrial wastewater, environmental water, and groundwater (Razali et al., 2012; Dolgushev et al., 2017). HBP is a kind of spherical polydisperse three-dimensional structure polymer with a linear chain part, branched part, and terminal part. Compared with ordinary dendritic branched molecules, they have similar physical and chemical properties and similar preparation methods, but HBPs have a more irregular shape, incomplete symmetry, and more terminal structures. The difference in the HBP structure lies in the molecular structure and the type and number of polymerized monomers.

In recent years, new hyperbranched polymers often have the properties of high solubility, controllable viscosity, and many end functional groups (Pearce et al., 2017; Chen et al., 2020). The adsorption and surface activity of hyperbranched materials have a wide application prospect in the field of ASP flooding wastewater treatment in oil fields (Shi et al., 2016). They can realize rapid sedimentation and oil–water separation through magnetic enrichment.

In this study, using the dispersion characteristics and magnetic properties of magnetic nanomaterials, the magnetic core is evenly dispersed in the material to ensure its magnetic response ability, and then the outer molecules are iteratively grafted to form a hyperbranched polymer. The rich end amino groups of the hyperbranched amide carbon chain have good demulsification performance, and the hyperbranched carbon chain can agglomerate the solid impurities and have flocculation performance. The magnetic hyperbranched polymer can break the stability and coalescence of ASP flooding wastewater, and the solid–liquid separation can achieve the purpose of water purification under the action of the external magnetic field.

## Experimental Section

### Materials

Tetraethyl silicate, ammonia, ethanol, carbon tetrachloride, polyaluminum chloride, cationic polyacrylamide (cationic degree 30), nano-Fe_3_O_4_ (50 nm), APTES, methanol, formic acid, formaldehyde, acetone, ethylenediamine, methyl acrylate, etc., all reagents (AR) used in the experiment were purchased from China Aladdin Reagent (Shanghai) Co. Ltd., and distilled water was self-made in the laboratory. Oilfield sewage was taken from crude oil separation sewage in a block of the Bohai oilfield (the suspended solid 143 mg/L, oil content 921.09 mg/L, turbidity 135 NTU, and zeta potential −47 MV).

### Synthesis of Fe_3_O_4_@SiO_2_

**FIGURE 1:**
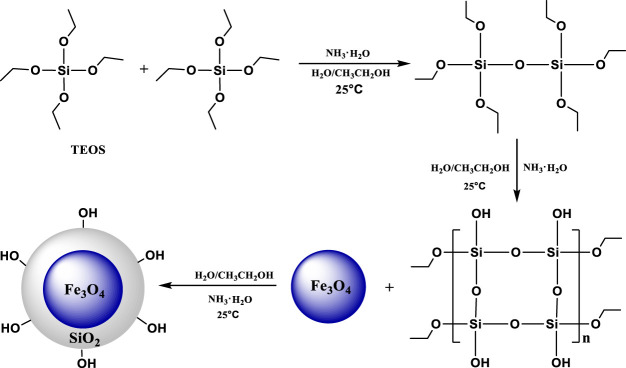
Synthesis of Fe_3_O_4_@SiO_2_.

Tetraethyl silicate (TEOS), with a chemical formula SiC_8_H_20_O_4_ and molecular weight 208.33, was used to hydrolyze in a mixed solution of ethanol and water, the generated SiO_2_ was deposited on the surface of the magnetic nano-Fe_3_O_4_ particles to form a spherical SiO_2_ coating (Figure 1).

$$\mathrm{Si}{\left(\mathrm{OC}H_{2}CH_{3}\right)}_{4}+H_{2}\mathrm{O-Si}{\left(\mathrm{OH}\right)}_{4}+C_{2}H_{5}\mathrm{OH}.$$

The silica shell thus formed contains silanol groups.

Fe_3_O_4_ powder was added to absolute ethanol solution for ultrasonic dispersion for 30 min, and the solution was transferred into a three-mouth flask. It was quantified to 500 ml (the ratio of ethanol to water was 4:1) and stirred evenly under certain temperature conditions. Then ammonia catalyst was added, and TEOS was added dropwise, and the coated particles can be obtained after reaction for a certain time.

In this experiment, excessive TEOS was used to improve the coating rate of Fe_3_O_4_. After the reaction, a large amount of absolute ethanol was used to wash and collect with magnetic sedimentation. Repeating this operation several times can remove the nonmagnetic SiO_2_ products and result in a high-purity magnetic core. This purification method was used to screen the products with excellent magnetic response ability, but the particle size of the magnetic core was larger, and the multicore Fe_3_O_4_ coated with SiO_2_ was more magnetic and easier to settle, which can be confirmed by transmission electron microscopy.

### Synthesis of Fe_3_O_4_@SiO_2_-NH_2_(FOSN)

**FIGURE 2:**
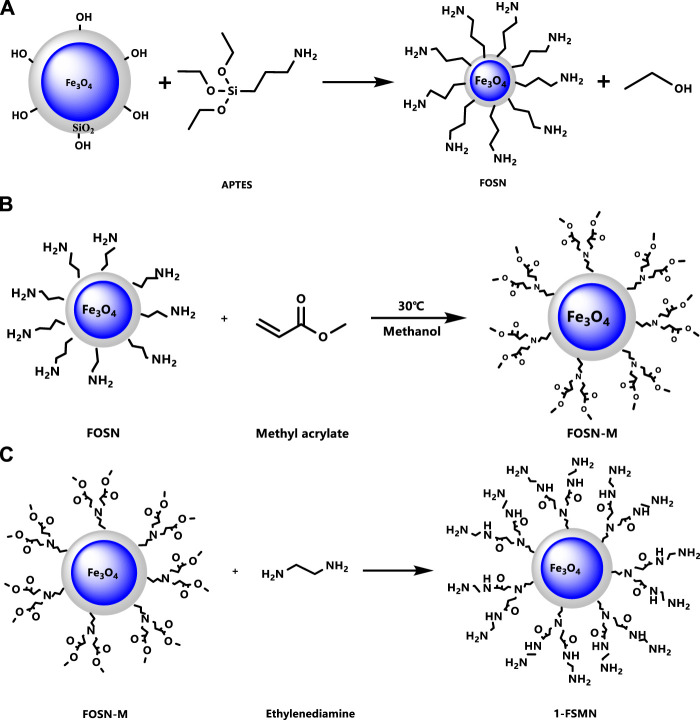
Synthesis of FOSN **(A)**, FOSN-M **(B)**, and 1-FSMN**(C)**.

As shown in Figure 2A, the ethoxy (-C_2_H_5_O) contained in APTES is hydrolyzed in aqueous solution and transformed into silicon hydroxyl (-SiOH). The silicon hydroxyl group of APTES can dehydrate and condense with the silicon hydroxyl group on the surface of nano Fe to obtain Fe_3_O_4_@SiO_2_-(CH_2_)_3_-NH_2_ (FOSN) and obtain the terminal-CH_2_-NH_2_ group. FOSN can form FS -CH_2_-N-(CH_2_-CH_2_-COOCH_3_)_2_ by Michael addition with methyl acrylate.

Fe_3_O_4_@SiO_2_ was added to 75% ethanol solution for ultrasonic dispersion for 20 min, and the solution was transferred into a three-mouth flask and stirred evenly at 78°C. Then Ammonia was added to it, and APTES was added dropwise at a uniform speed for 5 min and then it was kept to react for 6 h. After the reaction, the solution was washed with absolute ethanol, and the products were magnetically collected and dispersed in 30% methanol solution for 10 min to obtain the suspension of Fe_3_O_4_@SiO_2_-NH_2_ (FOSN). The surface amino density was estimated to be about 1.7–2.0 nm^−2^ by amino titration.

### Synthesis of HBP

With Fe_3_O_4_@SiO_2_-NH_2_ (FOSN) as the central core, the initial branched molecule FOSN-M was obtained by thorough Michael addition reaction with methyl acrylate, as shown in Figure 2B. Estimated from the median particle size and surface amino density of 1.7 nm^−2^, 1 mg of FOSN requires about 55 mg of methyl acrylate.

For the reaction, 1 g of FOSN was dissolved in 100 g of methanol; 55 g of methyl acrylate was added dropwise at a constant rate within 20 min, and the reaction was kept at 25°C for 24 h. The grafting rate after the reaction was about 70%, that is, 1 mg of FOSN can yield about 39 mg of FOSN-M.

Then 10 g FOSN-M was taken and dissolved in 30 g methanol. A constant-pressure funnel was used to drop 1 mol of ethylenediamine at a uniform rate within 10 min and was kept at 25°C for 24 h. FOSN-M carries out the amidation reaction of ester to obtain the first-generation branched molecule 1-FSMN, as shown in Figure 2C.

**FIGURE 3:**
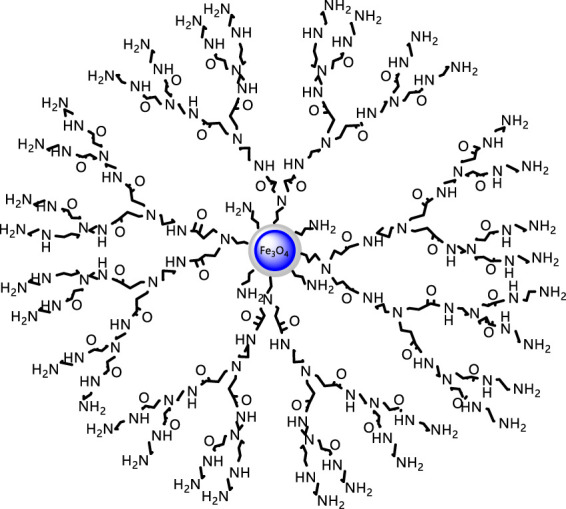
Synthesis of the third-generation branched molecule 3-FSMN.

The aforementioned Michael addition reaction and ester amidation reaction were repeated. In the Michael addition reaction, the molar ratio of ethylenediamine to methyl acrylate was 1:5, other conditions remained unchanged, and the second-generation hyperbranched molecule 2-FSMN can be obtained. Repeating the Michael addition reaction and the amidation reaction of the ester can give three generations of hyperbranched molecules (3-FSMN) (Figure 3).

#### Evaluation of the Synthesized Granularity

The particle size was measured using a laser particle size analyzer (Mastersizer 2000) from Malvern Panalytical, Germany. According to the Fraunhofer diffraction and Mie scattering theory, the particle size was analyzed by the diffraction of particles or the spatial distribution of scattered light (scattering spectrum). The test process was not affected by many factors such as temperature change, medium viscosity, sample density, and surface state. As long as the sample to be tested was evenly displayed in the laser beam, accurate test results can be obtained.

#### SEM/TEM

The electron microscope can scan the sample through a focused high-energy electron beam, excite, and collect physical information through the interaction between substances, and using computational imaging, the microscopic morphology of the sample after synthesis can be observed. A Hitachi SU8020 (EMAXevolution X-Max80/EX-270) magnetic material scanning electron microscope and a JEM-2100F transmission electron microscope (Japan Electronics Co., Ltd.) were used in the study to observe the surface morphology of magnetic nanoscale materials.

#### FT-IR

Fourier transform infrared spectroscopy (FT-IR) analyses of the hyperbranched polymer sample were performed using an IRTracer-100 (Shimadzu Corporation, Japan) device, and the LabSolutions IR analysis program was used to carry out data measurement and analysis. The samples scanned in the mid-infrared region of wavenumbers (400–4,000 cm^−1^) to record FT-IR spectra.

#### Thermogravimetric Analysis

A thermogravimetric analyzer was used to accurately measure the mass change and mass change rate of the tested sample. Through the thermogravimetric change, the physical and chemical changes of the sample during the heating process can be analyzed to infer its composition, thermal stability, and thermal decomposition. A DSC-Q200 differential scanning calorimeter (TA Company in the United States) was used.

#### VSM

The saturation magnetization curve was measured using a vibrating sample magnetometer (VSM), which was used for the testing of magnetic properties of magnetic materials. The saturation magnetization curve in this study was tested and plotted using a Lake Shore 7410 Vibrating Sample Magnetometer (Lake Company in the United States.

#### Yield

The calculation of product yield can help to verify the degree of a chemical reaction and judge the appropriate conditions of the reaction. The theoretical design product quality m_0_, the experimental product can be purified by magnetic sedimentation, washed with a large amount of solvent, and dried to obtain the product quality m.

$$\text{P}=\frac{m_{0}}{m}\times 100\%.$$

#### Oil Removal Rate

The wastewater used in the experiment was the wastewater after oil–water separation in the Bohai oilfield. The oil content of the sample solution was about 1,000 mg/L. The oil content was determined by the carbon tetrachloride extraction-spectrophotometer method, and the oil content c_1_ before treatment and the oil content c_2_ after treatment were measured, respectively. The oil removal rate R_0_ is calculated as follows:

$$R_{0}=\frac{c_{1}-c_{2}}{c_{1}}\times 100\%.$$

#### Removal Rate of Suspended Solids Containing Polymer

The suspended matter is filtered by the filter membrane suction method. The weight of the dried filter membrane is m_1_, the dilution ratio of the sample solution is a, the filtrate obtained by filtration is V_a_ ml, and the mass of the filter membrane is m_2_ after being fully dried at 100°C.

$$\text{S}=\text{a}\frac{m_{2}-m_{1}}{v_{a}},$$

$$R_{s}=\frac{S_{0}-S_{t}}{S_{0}}\times 100\%,$$

where R_s_ is the removal rate of suspended solids, S is the content of suspended solids, S_0_ is the content of suspended solids before treatment, and S_t_ is the content of suspended solids after treatment, a is the dilution multiple of the sample solution.

#### NTU

Suspended solids and colloids such as soil, silt, fine organic matter, inorganic matter, plankton, etc. can make the liquid turbid and produce a certain degree of turbidity. In the field of oilfield sewage treatment, turbidity is a significant indicator of water quality. In this experiment, the HACH 2100AN (NTU) turbidimeter (Hach Company) was used to measure the change in turbidity before and after treatment. The water sample for measuring turbidity needs to be measured within 24 h, and the water sample should be kept at room temperature and shaken before the measurement.

#### Zeta Potential

Zeta potential can quantitatively evaluate the stability of polymer sewage dispersion. The higher the absolute value of zeta potential, the more stable the sewage and the higher the treatment difficulty. In this experiment, the Malvern Zeta Potentiometer (Nano-ZS90) (Malvern Panalytical in Germany) was used to measure the change of zeta potential of the supernatant before and after treatment.

## Results and Discussion

### FT-IR

**FIGURE 4:**
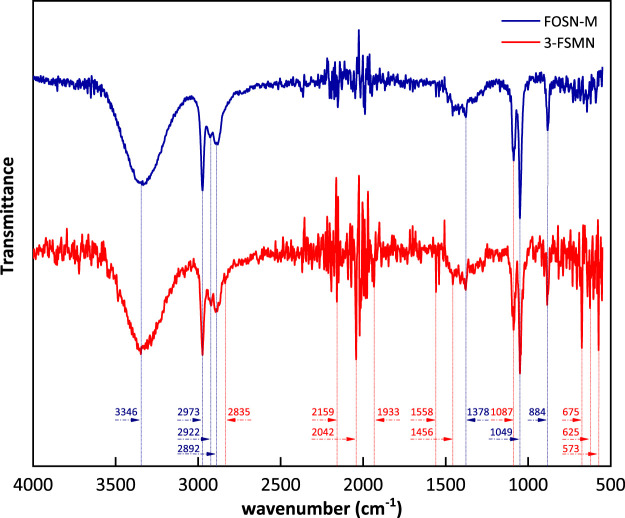
Infrared absorption spectra of FOSN-M and 3-FSMN.

The structures of FOSN-M and 3-FSMN have familial characteristics, corresponding to the structural similarity in the infrared absorption spectrum. The absorption spectrum is shown in Figure 4.

There are strong and wide absorption peaks at 346 cm^−1^, corresponding to the stretching vibration of N-H, secondary amide absorption peak, and -NH_2_ symmetrical stretching vibration absorption peak. Due to the difference in molecular structure, there is an induction effect, and the absorption peak of 3-FSMN has several wave number shifts. There are multiple methyl/methylene saturated carbon chain peaks at 2,973–2,892 cm^−1^, of which there are CH_3_ antisymmetric stretching vibration absorption peaks at 2,973 cm^−1^, 2,922 cm^−1^ is the antisymmetric stretching vibration absorption peak of -CH_2_- and 2,892 cm^−1^ is the C-H stretching vibration absorption peak, and the carbon chain structure of 3-FSMN repeats due to branching reaction, so the absorption intensity at 2,922 cm^−1^ and 2,892 cm^−1^ also increases. Moreover, due to the repeated appearance of branched structure CONH-, there is a weak and sharp absorption peak of C-H Fermi resonance affected by aldehyde group at 2,835 cm^−1^; 2,159 cm^−1^ corresponds to the sharp peak of silicone structure Si-H. The levels of these two compounds are very low, and there is a small amount of bare Si-H on the surface, which also shows that Si in H-Si-O is connected with organic matter. Multiple stretching vibration absorption peaks of C=O appear between 1934 and 2042 cm^−1^. The absorption peaks of 3-FSMN are more and the intensity is significantly enhanced. Compared with FOSN-M, there are more and more complex C=O structures. The peak value has different degrees of wave number shift, corresponding to its dendritic growth structure; 1,558 cm^−1^ is the C-N-H flexural vibration absorption peak of secondary amide. It can be seen that the increase in the secondary amide content the in 3-FSMN branched structure corresponds to the significant increase in absorption intensity. There is a weak C-N stretching vibration absorption peak of primary amide at 1,456 cm^−1^. Each generation of hyperbranched reaction is completed, the primary amine n, will appear on the corresponding branch chain, and its absorption intensity is also enhanced compared with FOSN-M; 1,378 cm^−1^ is -COO- weak symmetrical vibration absorption peak.

The antisymmetric stretching vibration strong absorption peak of Si-O-Si inorganic quartz structure appears at 1,087 cm^−1^, the strong absorption peak of Si-O-Si silicone appears at 1,049 cm^−1^, and the stretching vibration absorption peak of silicone Si-C appears at 884 cm^−1^, indicating that the silicon hydroxyl on the surface is silicone after being grafted and grown by APTES, and the silicon structure without hydroxyl on the surface does not participate in the reaction.

There are many small peaks near 750–700 cm^−1^, such as -NH_2_ torsional vibration of amide, out of plane rocking vibration of -NH_2_ and -NH, with weak intensity, but the peaks of 3-FSMN are significantly higher than those of FSON-M; 675 cm^−1^ is the in-plane bending vibration of amide O=C-N, and 625 cm^−1^ is the weak out of plane bending vibration absorption peak of O=C-N. The hyperbranched structure of 3-FSMN has three generations of -CONH branched structure, which has a very significant enhancement; 573 cm^−1^ is the -COO- out of plane swing near the CONH structure chain, with medium strength; obviously, it exists in the structure of 3-FSMN.

According to the analysis of the mid-infrared spectrum in Figure 4, FOSN-M is successfully connected with the organic amide ester structure on the outer surface of SiO_2_. While the family iterative reaction product 3-FSMN inherits the family structure, a large number of branched structures -CONH- appear. The primary amine N and secondary amine NH of each generation of intermediate connecting atoms designed theoretically also exist in the molecular structure, and the relevant absorption peaks of -CONH- and -NH_2_ are also significantly enhanced. It is consistent with the expectation of the experimental molecular design.

The infrared absorption spectra of 1-FSMN, 2-FSMN, and 3-FSMN are consistent. The absorption peak intensities of -CONH branched structure, primary amine n, secondary amine NH, and NH_2_ are 3-FSMN > 2-FSMN > 1-FSMN, which is in line with the molecular design of family iterative growth.

### SEM/TEM

#### TEM Characterization of Fe_3_O_4_@SiO_2_ Magnetic Nucleus

**FIGURE 5:**
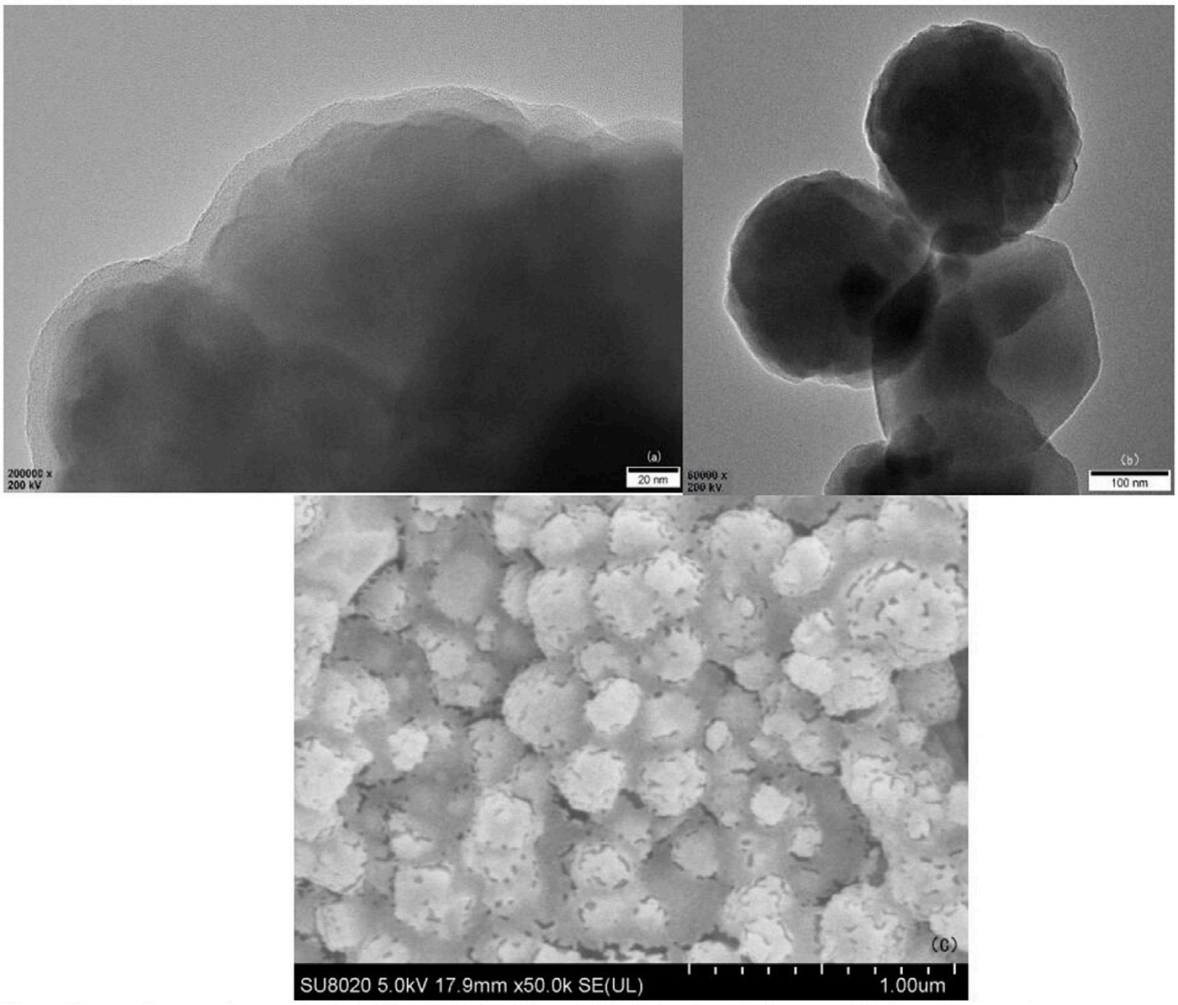
Magnetic core Fe_3_O_4_@SiO_2_ TEM **(A)**, **(B)**, and SEM **(C)** of 3-FSMN material after gold spraying treatment.

In Figures 5A,B obtained by TEM, the morphology shows that in the products collected by magnetic sedimentation, multiple Fe_3_O_4_ particles and SiO_2_ structure form a spherical central magnetic core Fe_3_O_4_@SiO_2_, Figure 5A shows that its surface is successfully coated with a SiO_2_ layer of tens of nanometers. This SiO2 structure is produced by TEOS condensation, with silicon hydroxyl groups on the surface.

#### SEM Characterization of 3-FSMN

The 3-FSMN material was dried at 10°C and made into thin sheets. Because the hyperbranched organic matter in the outer layer of the material was not conductive, gold spraying SEM imaging was required. As can be seen from Figure 5C, a layer of the hyperbranched polymer was successfully synthesized on the surface of Fe_3_O_4_@SiO_2_.

### Influencing Factors of Fe_3_O_4_ Magnetic Core Particle Size

The particle size of the Fe_3_O_4_@SiO_2_ core affects its magnetic response performance, so it is necessary to find a suitable particle size. In the preparation process, repeated ethanol washing and magnetic sedimentation collection can obtain high-purity central magnetic core Fe_3_O_4_@SiO_2_. The median particle size of the magnetic core with rapid magnetic sedimentation obtained by this method is 285 nm.

#### The Effect of Reaction Time

Fe_3_O_4_ powder was added to anhydrous ethanol solution for ultrasonic dispersion for 30 min, and the solution was transferred into a three-necked flask (the ratio of ethanol to water is 4:1) and was stirred evenly at 30°C. Catalyst ammonia was added, as well as 1% TEOS was added dropwise, and the solution was allowed to react for 1, 2, 3, 4, 5, and 6 h, respectively.

**FIGURE 6:**
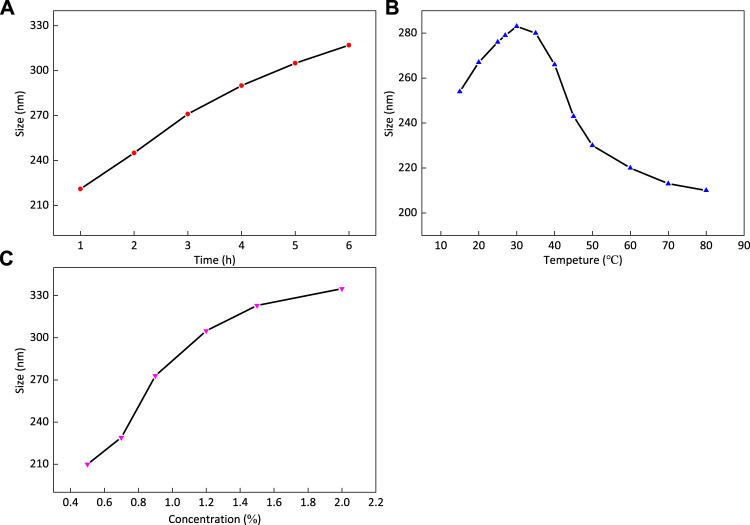
Particle size of central magnetic core Fe_3_O_4_@SiO_2_ is affected by reaction time **(A)**, reaction temperature **(B)**, and reactant concentration **(C)**.

As shown in Figure 6A, other reaction conditions remain unchanged. With the extension of reaction time, the average particle size of the product increases and then tends to decrease. The reason is that the number of outer cladding layers increases and the particle size increases with the passage of time. At the same time, the total amount of TEOS is limited, and the growth rate becomes smaller. It can be seen that the reaction time required to reach the median particle size of 285 nm is about 4 h.

#### The Effect of Reaction Temperature

Fe_3_O_4_ powder was added into anhydrous ethanol solution for ultrasonic dispersion for 30 min, and the solution was transferred into a three-mouth flask, with the ratio of ethanol to water being 4:1. The solution was stirred evenly under different temperature conditions, respectively. To the solution, catalyst ammonia was added, and then 1% TEOS was added dropwise, and then was left to react for 4 h.

As shown in Figure 6B, other reaction conditions remain unchanged. With the increase in reaction temperature, reaction time, and dosage conditions, the average particle size of the product first increases and then decreases, and the trend decreases with the increase in temperature. Theoretically, with the increase in temperature, the reaction rate accelerates, and the number of coating layers should increase, resulting in an increase in particle size. The reason is that the target product of this experiment is magnetic core microspheres, and the total amount of TEOS is limited. With the increase in temperature, the hydrolysis rate of TEOS increases, the resulting silicon layer thickens and the particle size increases; after exceeding 30°C, the hydrolysis rate of TEOS further increased, and more SiO_2_ microspheres with uncoated magnetic core were formed and washed, resulting in the decrease in TEOS involved in coating and the average particle size decreased slightly. The problem of hollow SiO_2_ microspheres produced by too fast TEOS reaction often appears in the research. The reaction temperature required for the median particle size to reach 285 nm is about 30°C.

#### The effect of Reactant Concentration

Fe_3_O_4_ powder was added to anhydrous ethanol solution for ultrasonic dispersion for 30 min, and the solution was transferred into a three-mouth flask; the ratio of ethanol to water was 4:1. The solution was stirred evenly at 30°C, and catalyst ammonia was added to it, and then some TEOS was added dropwise and kept to react for 4 h. As shown in Figure 6C, when the TEOS dosage concentration increases and the reaction time and temperature remain unchanged, the average particle size of the product increases and then decreases. The reason is that the hydrolysis of TEOS has a certain speed limit under certain time and temperature conditions. When the concentration is low, the speed of SiO_2_ formation does not reach the limit, so the amount of SiO_2_ increases with the increase in dosage; when the concentration reaches a limit, the rate of SiO_2_ formation reaches the limit, and the growth rate of particle diameter will slow down. It is not difficult to see that the TEOS concentration of 1% can meet the needs of this experiment. This study needs a good magnetic response and small particle size. The purpose of the particle size experiment is to make the reaction conditions as close as possible to the conditions required by the median particle size obtained by magnetic separation, to improve the yield.

This part of the experiment was actually affected by the purification experimental method. In this study, magnetic collection is used, resulting in the removal of most Fe_3_O_4_ single-core particles with small particle size in the magnetic cleaning stage. Because the magnetic core is small, the shielding effect relative to the silicon layer with the same thickness is enhanced, resulting in the decline of their magnetic performance. The small-scale agglomerated multicore Fe_3_O_4_ has strong magnetic properties and is left in the system, which can be proved from the TEM image.

### VSM

**FIGURE 7:**
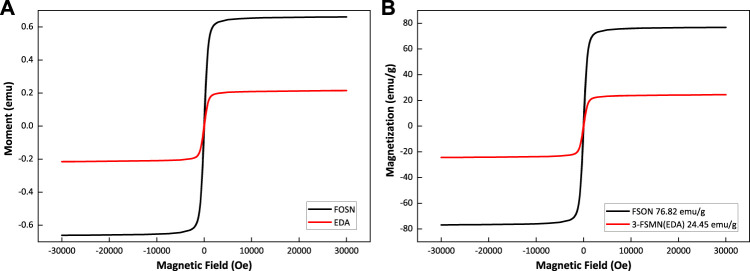
Magnetization properties of magnetic amino-terminal core FOSN and third-generation hyperbranched molecule 3-FSMN.

The magnetization properties of magnetic amino-terminal core FOSN and third-generation hyperbranched molecule 3-FSMN are shown in Figure 7.

Figure 7 shows that the FOSN magnetic core and the third-generation hyperbranched molecule 3-FSMN are ferromagnetic materials. The saturation magnetization of 3-FSMN decreases because of the growth of hyperbranched molecules on its surface. FSON is 76.82 emu/g, and 3-FSMN is reduced to 24.45 emu/g, but it still has sufficient magnetic response capacity.

### Thermogravimetry

**FIGURE 8:**
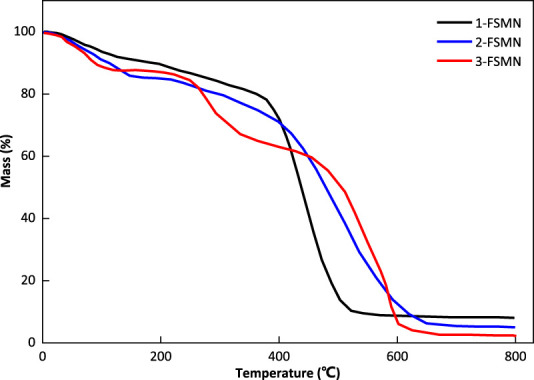
Thermogravimetric curves of 1-FSMN, 2-FSMN, and 3-FSMN materials.

The curves of 1-FSMN, 2-FSMN, and 3-FSMN show (Figure 8) that the main structural units in the front section of the thermogravimetric loss curve of the FSMN family are -CH_2_-NH_2_ substances, which exist at the ends of the three substances and the upper ends of their branched structures, and the structural units lost in the rear section are the main branched structure -CH_2_N-(CH_2_-CONH-CH_2_)_2_. At the same time, the decomposition speed of ester functional groups is faster after reaching the decomposition temperature; when the temperature exceeds 600°C, thermal decomposition occurs violently inside the molecule, resulting in a sharp drop in the curve. The remaining material is the ferromagnetic core coated with SiO_2_.

Figure 8 shows that the thermal decomposition before 600°C will start from the end of the outer layer, 1-FSMN shows a relatively smooth single-stage decomposition trend, 2-FSMN shows an approximate two-stage decomposition trend, and 3-FSMN shows an obvious three-stage decomposition trend. This decomposition trend is in line with the family iterative growth mode of amino-terminal branched structure. It shows that there are three generations of repeated branched structures in 3-FSMN. And there are SiO_2_ cores resistant to 800°C, which is in line with the expectation of hyperbranched molecular design.

#### Determination of Optimum Reaction Conditions

Appropriate conditions for synthesis experiments can be intuitively selected by evaluating the yield of hyperbranched molecules.

### Yield of 1-FSMN

Taking the reaction time, reaction temperature, the mass ratio of ethylenediamine to FOSN-m, and the amount of dispersant methanol as the influencing factors, the orthogonal experiment was carried out according to table L9 (34), the influence of each influencing factor on the synthesis of 1-FSMN branched molecules were determined, and the most suitable conditions for the synthesis of 1-FSMN branched molecules were obtained. In HBP synthesis, it is estimated that the mass of methyl acrylate accounts for the vast majority of the molecular weight of FOSN-m, so the molar weight of FOSN-M is approximately calculated by the molecular weight of methyl acrylate.

This part of the calculation will not be described in detail, but it should be noted that this proportion is closely related to the surface physical and chemical properties of the central magnetic core. Repeated experiments need to redetermine the surface properties before calculation. In order to ensure the consistency of the experiment, all subsequent synthetic materials should use the same batch of measured and recalculated magnetic core.

Table 1A shows the orthogonal experimental factors, and Table 1B shows the experimental results. In the table, A represents reaction time (H); B denotes reaction temperature (°C); C is the molar ratio of EDA to FOSN-M; and D is the amount of dispersant methanol (% wt), the percentage of dispersant methanol in the total mass of the reaction solution.

**TABLE 1A:** 1-FSMN orthogonal experiment table.

Factor\level	1	2	3
A	12	24	36
B	25	35	45
C	0.25	0.125	0.04
D	20	30	40

**TABLE 1B:** Orthogonal experimental data of 1-FSMN branched molecule synthesis.

Test group	A	B	C	D	Yield
1	A1	B1	C1	D1	85.3
2	A1	B2	C2	D2	86.9
3	A1	B3	C3	D3	85.1
4	A2	B1	C2	D3	90.6
5	A2	B2	C3	D1	84.3
6	A2	B3	C1	D2	84.7
7	A3	B1	C3	D2	90.7
8	A3	B2	C1	D3	80.5
9	A3	B3	C2	D1	89.8
K1	257.3	266.6	250.5	259.4	—
K2	259.6	251.7	267.3	262.3	—
K3	261	259.6	260.1	256.2	—
R	3.7	14.9	16.8	6.1	—
G	1.00	4.03	4.54	1.65	—

The reading range in the table is 3.7, 14.9, 16.8, and 6.1, respectively, and the ratio G is 1, 4.03, 4.54, and 1.65, respectively. Then the influence degree of the four factors on the yield of synthetic products is C > B > D > A, in which the G value of CBD is greater than 1.5, which is a significant influencing factor. It can be seen that the largest influencing factor is the ratio of the amount of ethylenediamine and FOSN-M, and the influence degree of reaction temperature is the second. The least influence factor on the reaction is the reaction time.

The optimum synthesis conditions obtained by orthogonal experiment are A3B1C2D2, that is, the reaction temperature is 25°C, the reaction time is 24 h, the amount ratio of EDA to MA (FOSN-m) is = 1:8, and the amount of dispersant methanol is 30%. At this time, the yield can reach 90.6%.

#### Influence of the Ratio of Ethylenediamine to FOSN-M on the Yield of 1-FSMN Branched Molecules

**FIGURE 9:**
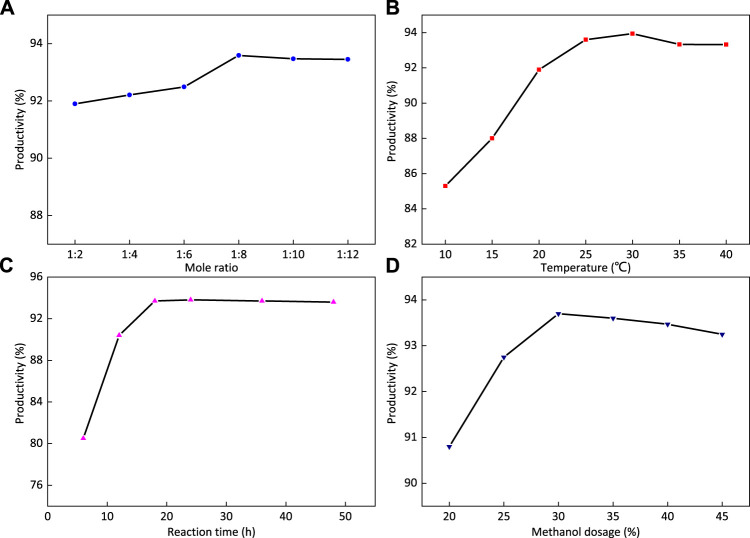
Effects of reaction time **(A)**, reaction temperature **(B)**, the ratio of ethylenediamine to FOSN-M **(C)**, and the amount of dispersant methanol **(D)** on the yield of 1-FSMN.

The reaction conditions are as follows: the reaction time is 24 h, the reaction temperature is 25°C, the amount of dispersant methanol is 30%, and the effect of the ratio of the amount of ethylenediamine to FOSN-M on the yield of the 1-FSMN branched molecule is studied. The experimental results are shown in Figure 9A.

Figure 9A shows that with the increase in the ratio of ethylenediamine to FOSN-M, the yield first increases and then decreases. The inflection point is when the ratio of ethylenediamine to methyl acrylate is 1:8. The reason is that the Michael addition reaction of ethylenediamine and methyl acrylate can be fully reacted at room temperature, and the increase in the amount of methyl acrylate will speed up the reaction and make it easier to remove. When it exceeds 1:8, the reaction rate will not be significantly accelerated, so the ratio of ethylenediamine to methyl acrylate used in this experiment is 1:8.

#### Influence of Branching Reaction Temperature on the Yield of 1-FSMN Branched Molecules

**FIGURE 10:**
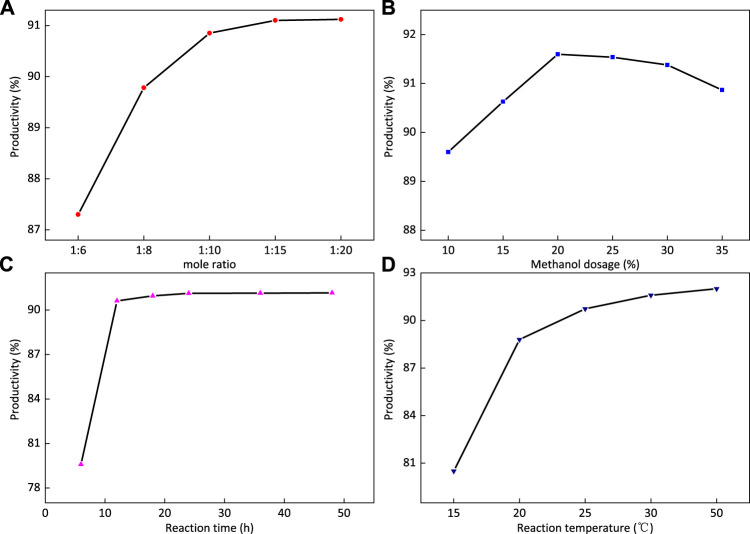
Effects of the ratio of reactants **(A)**, methanol dosage **(B)**, reaction time **(C)**, and reaction temperature **(D)** on the yield of 2-FOSN hyperbranched molecules.

The reaction conditions are as follows: the reaction time is 24 h, the amount of dispersant methanol is 30%, and n (EDA):n (MA) = 1:8. The effect of reaction temperature on the yield of the 1-FSMN branched molecule is studied. The experimental results are shown in Figure 10B.

Figure 9B shows that the yield of 1-FSMN branched molecules first increases and then decreases with the increase in temperature. This part of the reaction competes with the self-polymerization of methyl acrylate and intramolecular amidation reaction. At low temperatures, the Michael addition reaction occupies an absolute advantage. The increase in temperature can not only increase the reaction rate but also reduce the competitive advantage of the Michael addition reaction.

When the reaction temperature was 30°C, the self-polymerization and intramolecular amidation level of methyl acrylate were the lowest, and the yield of 1-FSMN branched molecule reached the highest value, up to 93.9%. Therefore, the reaction temperature was determined to be 30°C.

#### Effect of Reaction Time on Yield of Synthesis of 1-FSMN Branched Molecules

In the experiment, it was found that although the reaction time had the least significant effect on the yield of 1-FSMN branched molecules, if the reaction time was long enough, it was necessary to find this time node to minimize its influencing factors. The reaction conditions are as follows: the reaction temperature is 30°C, the amount of dispersant methanol is 30%, and n (EDA):n (MA) = 1:8. The effect of reaction time on the yield of 1-FSMN branched molecules is studied. The time intervals are 6, 12, 18, 24, 36, and 48 h, respectively. The experimental results are shown in Figure 9C.

Figure 9C shows that with the extension of reaction time, the yield of the product increases first and then becomes stable, in which it does not increase at 18 h and the yield does not change. The reason is that when the reaction time is short, Michael addition does not fully occur. When the reaction time reaches the point of 18 h, the Michael addition reaction has fully occurred, the reaction time continues to increase, the yield reaches 93.7% and does not increase any more, and the formation reaction of 1-FSMN hyperbranched molecules is over. Therefore, the reaction time was determined to be 18 h.

#### Effect of Dispersant Methanol Addition on Yield of Synthesized 1-FSMN Branched Molecules

The reaction conditions were as follows: reaction temperature 30°C, reaction time 18 h, and n (EDA):n (MA) = 1:8. The effect of the amount of dispersant methanol on the yield of 1-FSMN branched molecules was studied. Figure 9D shows that with the increase in the amount of dispersant methanol, the yield first increases and then decreases. When the amount of methanol is 30%, the yield reaches the highest value of 93.7% and then decreases with the increase in the amount of methanol.

A small amount of methanol solvent cannot fully activate all reactants. With the increase in the solvent amount, all reactants enter the reaction system, and the yield increases. After more than a 30% increase, the solvation effect is dominant, which hinders the movement of reaction equilibrium to the product direction, and the yield decreases slightly. Therefore, the dosage of dispersant methanol should be 30%. According to the yield, the synthesis conditions are as follows: the reaction time is 18 h, the reaction temperature is 30°C, the mass ratio of ethylenediamine to methyl acrylate (FOSN-m) = 1:8, the amount of dispersant methanol is 30%, and the yield is 93.7%.

### Yield of Progeny Reaction

When 1-FSMN reacts with excess methyl acrylate (MA), the dosage range can be estimated by the aforementioned method. The reaction product is 1-FSMN-M, and its relative molecular weight is determined after purification. The same method was used to treat the offspring intermediates such as 2-FSMN-M and 3-FSMN-M.

#### Orthogonal Experiment of 2-FSMN Hyperbranched Molecular Synthesis

Taking the reaction time, reaction temperature, the ratio of 1-FSMN-M branched molecule to ethylenediamine, and the amount of dispersant methanol as the influencing factors, the orthogonal experiments were carried out according to the L9 (34) table to determine the effects of various reaction conditions on the synthesis yield of 2-FSMN hyperbranched molecules, and the most suitable synthesis conditions for the synthesis of 2-FSMN hyperbranched molecules were obtained.

Table 2A is the orthogonal experimental factor, and Table 2B is the experimental result. In the table, A is the reaction time (h); B is the reaction temperature (°C); C is the molar ratio of 1-FOSN-M to EDA; and D is the amount of methanol added as dispersant (%).

**TABLE 2A:** 2-FSMN orthogonal experiment table.

Factor\level	1	2	3
A	12	24	36
B	25	35	45
C	0.25	0.125	0.04
D	20	30	40

**TABLE 2B:** Orthogonal experimental data of 2-FSMN branched molecule synthesis.

Test group	A	B	C	D	Yield
1	A1	B1	C1	D1	85.5
2	A1	B2	C2	D2	88.8
3	A1	B3	C3	D3	89.7
4	A2	B1	C2	D3	88.9
5	A2	B2	C3	D1	91.6
6	A2	B3	C1	D2	84.1
7	A3	B1	C3	D2	90.5
8	A3	B2	C1	D3	85.3
9	A3	B3	C2	D1	90.8
K1	264	264.9	254.9	267.9	—
K2	264.6	265.7	268.5	263.4	—
K3	266.6	264.6	271.8	263.9	—
R	2.6	1.1	16.9	4.5	—
G	2.36	1.00	15.36	4.09	—

The readout ranges in the table are 2.6, 1.1, 16.9, and 4.5, respectively, and the ratios G are 2.36, 1, 15.36, and 4.09, respectively, so the influence degree of the four factors on the yield of synthetic products is C > D > A > B; among them, the g value of CDA is greater than 1.5, which is a significant influencing factor. The largest influencing factor is the ratio of the amount of 1-FOSN-M branched molecule to ethylenediamine, followed by the amount of methanol solvent, and the reaction time ranks third.

The results showed that the optimum synthesis conditions were as follows: A2B2C3D1, that is, the reaction temperature was 35°C, the reaction time was 24 h, the ratio of 1-FOSN-M to EDA was 1:15, the amount of dispersant methanol was 20%, and the yield was 91.6%.

#### Yields of 2-FSMN Hyperbranched Molecules

##### Influence of the Ratio of the Amount of 1-FOSN-M Branched Molecules to Ethylenediamine on the Yield of 2-FOSN Hyperbranched Molecules

The reaction conditions were as follows: the reaction time was 24 h, the reaction temperature was 35°C, the amount of dispersant methanol was 20%, and the effect of the ratio of the amount of 1-FOSN-M branched molecule to ethylenediamine on the yield of the 2-FOSN hyperbranched molecules was studied.

Figure 10A shows that the yield increases first and tends to be stable with the increase in the mass ratio of substances. When the mass ratio of additives is 1:15, the maximum yield can be reached. The reason is that the end group amidation of ethylenediamine and 1-FOSN-M branched molecules can completely react at room temperature. The increase in excess ethylenediamine will accelerate the reaction speed, enhance the competitiveness of the main reaction, and hinder the occurrence of side reactions such as intramolecular amidation. Therefore, the mass ratio of 1-FSMN-M to ethylenediamine used in this test is 1:15.

##### Effect of the Amount of Dispersant Methanol on the Yield of 2-FOSN Hyperbranched Molecules

The reaction conditions are as follows: the reaction time is 24 h, the reaction temperature is 35°C, and the ratio of 1-FSMN-M branched molecule to ethylenediamine is 1:15. The effect of the amount of dispersant methanol on the yield of the 2-FOSN hyperbranched molecule is studied. The results are shown in Figure 10B. Figure 10B shows that with the increase in the amount of dispersant methanol, the yield first increases and then decreases. When the amount of methanol is 20%, the yield reaches the highest value and then increases with the amount of methanol, and the yield shows a downward trend.

The reason is that when the amount of methanol is small, the number of free molecules available for reaction in the solution is small, the reaction speed is slow, and the yield is low. When the amount of methanol increases, the solvation effect decreases the relative concentration of reactants, resulting in a decrease in reaction speed and yield. Therefore, the dosage of dispersant methanol is 20%.

##### Effect of Reaction Time on the Yield of 2-FOSN Hyperbranched Molecules

The reaction conditions are as follows: the reaction time is 24 h, the reaction temperature is 35°C, and the weight ratio of 1-FSMN-M branched molecule to ethylenediamine is 1:15. The effect of synthesis reaction time on the yield of the 2-FOSN hyperbranched molecule is studied. The experimental results are shown in Figure 10C. Figure 10C shows that with the extension of reaction time, the yield of the product increases first and then becomes stable, it does not increase at 24 h, and the yield does not change. The reason is that the amidation of ethylenediamine and terminal ester group occurs very rapidly in a short reaction time, which has exceeded 90% in 12 h. When the reaction time reaches the point of 24 h, the reaction has fully occurred, the reaction time continues to increase, and the yield reaches the maximum without increasing. The reaction time was 24 h, and the formation reaction of 2-FOSN hyperbranched molecules was over.

##### Effect of Reaction Temperature on the Yield of 2-FOSN Hyperbranched Molecule

The reaction conditions are as follows: the reaction time is 24 h, the ratio of 1-FSMN-M branched molecule to ethylenediamine is 1:15, and the amount of methanol solvent is 20%. The effect of synthesis reaction temperature on the yield of the 2-FOSN hyperbranched molecule is studied. The experimental results are shown in Figure 10D. Figure 10D shows that with the increase in reaction temperature, the yield of the 2-FOSN hyperbranched molecule first increases and then stabilizes, and the growth rate slows down at 25°C. During the experiment, it is found that when the temperature exceeds 30°C, the separation of products tends to be difficult. Combined with the literature, it is found that when the temperature increases, the degree of reverse reaction and side reaction will also increase. When the reaction temperature reaches 35°C, the reaction temperature can be determined as 35°C.

The aforementioned experiments show that the reaction conditions for the synthesis of 2-FSMN hyperbranched molecules are determined as follows: the reaction time is 24 h, the reaction temperature is 35°C, the amount of methanol solvent is 20%, the ratio of 1-FSMN-M branched molecules to ethylenediamine is 1:15, and the yield is 91.6%. The reaction conditions of branching reaction are very close between different offspring, and the yield difference is small under the same conditions, so the offspring also choose the same reaction conditions to prepare 3-FSMN, 4-FSMN, etc.

##### Water Treatment Performance

The sewage used in the experiment is the sewage after oil–water separation in the Bohai oilfield. The sewage sample solution contains a poly-suspension of 143 mg/L, oil content of 921.09 mg/L, turbidity of 135 NTU, and zeta potential of -47 MV. The system is relatively stable and difficult to be broken by physical means. The organic matter in the sewage is mainly residual oil phase and polymer, the suspended matter is solid particles such as clay and rock debris, and most surfactant molecules also exist at the interface of each phase.

For hyperbranched molecules, 1-FSMN, 2-FSMN, 3-FSMN, and 4-FSMN are selected for performance experiments and compared with the terminal quaternary ammonium salt (QAS) of each hyperbranched molecule. They are named 1-FSMN-Q, 2-FSMN-Q, 3-FSMN-Q, and 4-FSMN-Q. The terminal quaternary ammonium degree estimated by the conductivity enhancement method is about 20%. At the same time, the corresponding terminal quaternary ammonium salt is selected for experiments.

**FIGURE 11:**
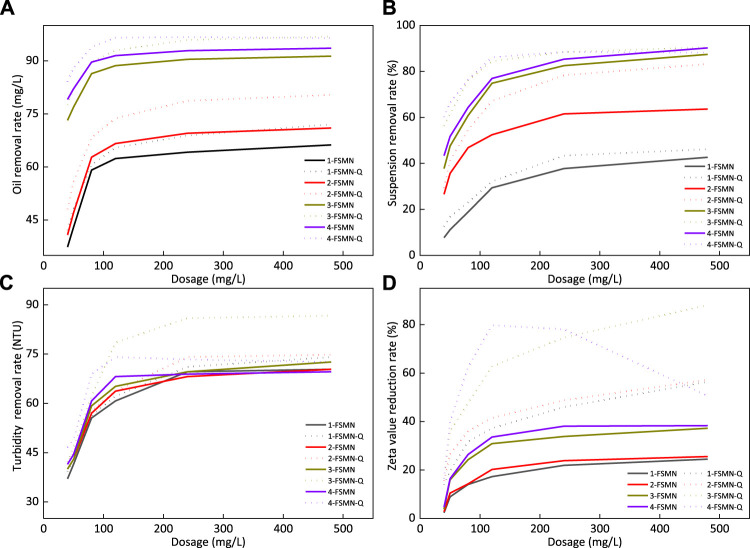
Oil removal rate **(A)**, suspended solids removal rate **(B)**, turbidity removal rate **(C)**, and zeta potential reduction rate **(D)** of FSMN family and its quaternary ammonium salt.

The experimental condition is room temperature 25°C, the reagent was added, stirred evenly, allowed to stand for 1 min, then centrifuged for 5 min, and took the upper liquid for determination. The experimental results are shown in Figure 11.

#### Oil Removal Rate

Figure 11A shows that the improvement of branching algebra will improve the demulsification ability of branched polyamide amine structure. The oil removal rate of 3-FSNM can reach 91.4% and that of 4-FSNM can reach 93.6%. The oil removal capacity of the corresponding quaternary ammonium salt is stronger than that of the unmodified hyperbranched molecule FSNM, and the performance of 4-FSNM-Q is stronger than that of 3-FSNM-Q, but the oil removal capacity is almost the same when the dosage is sufficient, and the oil removal rate can reach 97%. Hyperbranched polyamide structure can adsorb and agglomerate emulsified oil droplets into large oil droplets and separate them from the system. However, the surface of oil droplets in oilfield sewage is usually negatively charged, especially the oil droplets with small particle size, which have relatively strong electrostatic repulsion and are difficult to agglomerate.

With the assistance of electrostatic adsorption, the terminal quaternary ammonium salt of FSMN-Q can better agglomerate fine oil droplets and obtain a better oil removal effect. Structurally, if the quaternization degree is the same, the hyperbranched molecular end of high algebra FSMN-Q has more quaternary ammonium radicals, and the demulsification ability increases with the algebra. It can also be found in Figure 11A that the treatment effect of 4-FSMN-Q is much higher than that of its parent quaternary ammonium salt at low concentration, but at high dosage, the electrical reversal leads to the reduction of its oil removal effect, which is finally slightly lower than that of 3-FSMN-Q.

#### Removal Rate of Suspended Solids

Figure 11B shows that the improvement of branching algebra will significantly improve the adsorption and flocculation capacity of FSMN molecules. 3-FSNM can achieve an 87.4% suspended solids removal rate and 4-FSNM can achieve a 90.2% suspended solids removal rate. The removal capacity of the corresponding quaternary ammonium salt suspension is stronger than that of the unmodified hyperbranched molecule FSNM, and the difference is particularly significant under the condition of low dosage. 4-FSNM-Q is stronger than 3-FSNM-Q at low dosage, but it will decrease slightly with the dosage after reaching 88%, which is smaller than that of the unmodified 4-FSNM. 3-FSNM-Q can achieve the maximum suspended solids removal rate of 90.5%.

The suspended solids are mainly composed of clay, rock debris, and other solid phases. These solid particles are negatively charged and can settle the suspended solids well under the action of FSMN hyperbranched molecule adsorption bridging and electrostatic attraction. It can be seen intuitively in Figure 11B that the effect of FSMN hyperbranched molecules in the offspring in removing suspended solids at low concentration is stronger than that of the parent. The treatment effect of quaternary ammonium modified FSMN-Q is stronger than that of FSMN due to electrostatic attraction.

However, at higher concentrations, the treatment effect of 3-FSMN and 4-FSMN is similar to that of quaternary ammonium salt. There are two main reasons. The suspended solids are sensitive to viscosity. 3-FSMN-Q and 4-FSMN-Q increase the system viscosity at high dosage, resulting in the slowdown of the treatment effect with dosage. The adsorption bridging effect of hyperbranched molecules accounts for the main factor. Finally, the treatment effect is close to that of 3-FSMN and 4-FSMN hyperbranched molecules. The treatment effect of 4-FSMN-Q decreases slightly due to electrical turnover. The removal rate of suspended solids of 4-FSMN and 3-FSMN-Q can reach 90.3%.

#### Turbidity(NTU)

Figure 11C shows that at a lower dosage, the turbidity removal rate increases with the branching algebra, and 4-FSMN is the strongest. After exceeding a certain dosage, the turbidity removal capacity of 4-FSMN decreases significantly. The corresponding quaternary ammonium salt also has this feature. The effect of 4-FSMN-Q is significant at a low dosage. After reaching the maximum removal rate, the removal rate decreases to a certain extent with the further increase in dosage. 3-FSMN can achieve the maximum turbidity removal rate of 86.4%. The main contributors to the turbidity of polymer containing ASP flooding wastewater used in this experiment are oil droplets, clay, and rock cuttings, and some colored surfactants are mostly adsorbed on the surface of oil droplets or solid particles. Therefore, the turbidity reduction ability of high algebra FSMN with strong oil removal ability and suspended solids removal ability is stronger. At a lower dosage, 4-FSMN-Q is stronger than 3-FSMN-Q, and its corresponding FSMN hyperbranched molecules. With the increase in dosage, the viscosity of the system increases, and the electrical inversion of 4-FSMN-Q molecules after treatment leads to the deviation between the actual and theoretical treatment effect. After exceeding a certain dosage, the treatment effect is almost no longer improved. For the sewage in this experiment, the relative treatment effect of 3-FSMN and its quaternary ammonium salt is the best. Among them, 3-FSMN-Q can reduce the turbidity by 86.6%.

#### Zeta Potential

Figure 11D shows that FSMN hyperbranched molecules can reduce the zeta potential value of this polymer containing wastewater to a certain extent, and the ability to reduce the zeta potential value increases slightly with the increase in branching algebra. The maximum reduction value of 4-FSMN is 38.2%, but this reduction effect is actually indirect grounding, which reduces the content of negatively charged rock cuttings and oil droplets in the system through demulsification flocculation sedimentation. It is not directly neutralized; so after reaching a certain reduction rate, the zeta potential value hardly changes, which is just in line with the previous experimental phenomenon.

Quaternary ammonium salt FSMN-Q has a more significant reduction rate of zeta potential, and its effect is stronger than that of the FSMN protomolecule. Similarly, the content of negatively charged rock cuttings and oil droplets in the system is reduced through demulsification flocculation sedimentation, and the quaternary ammonium ion at its end is electrically neutralized with negatively charged particles, greatly reducing the zeta potential. However, for sewage, after adding too much cationic material, its electrical property may be reversed. In this experiment, it can be observed that the electrical property of 4-FSMN-Q is reversed at a higher concentration, and the treatment effect becomes worse, which is reduced to 51.1% at the dosage of 480 mg/L. The branching degree of 3-FSMN-Q is less than that of 4-FSMN-Q, and the relative content of quaternary ammonium at the end is also lower than that of 4-FSMN-Q, and the treatment effect is relatively inferior at a lower dosage. However, the addition of 480 mg/L also failed to reverse the electrical property of the sewage, and the reduction rate of the zeta potential value can reach 88.1%.

Based on the experimental phenomena in Figure 11, it is not difficult to see that the water treatment capacity characteristics of magnetic hyperbranched molecule FSMN are similar to those of cationic polypropionamide. To determine the optimal dosage of the treatment agent, the influence of viscosity change in the sewage system needs to be considered. For cationic modified FSMN-Q, the change in the sewage zeta potential also needs to be observed. Based on the aforementioned results, for the sewage treatment used in this experiment, 3-FSMN and its quaternary ammonium salt 3-FSMN-Q can meet the treatment requirements. At the same time, in order to further enhance the treatment effect and reduce the treatment cost, inorganic flocculants such as polyaluminum chloride (PAC) can also be compounded to enhance their adsorption and precipitation net capture capacity.

##### Magnetic Sedimentation Performance Experiment

The wastewater used in the experiment was the wastewater after oil–water separation in the Bohai oilfield, with suspension 143 mg/L, oil content 921.09 mg/L, turbidity 135 NTU, and zeta potential −47 mV. The experimental conditions were room temperature 25°C; adding the agent and stirring evenly, one group settled naturally under gravity for 5 min and the other group settled under the lateral 90° neodymium magnet magnetic field for 5 min. The liquid phase after sedimentation was taken to measure the oil removal rate, suspended solids, and turbidity changes. The hyperbranched treatment agent was compounded with 3-FSMN and 3-FSMN-Q with PAC (mass ratio FSMN:PAC = 1:1), and the experimental control group was compounded with cationic polyacrylamide and polyaluminum chloride (mass ratio CPAM:PAC = 3:10), and the magnetic sedimentation groups were named 3-FSMN(mag.) and 3-FSMN-Q (mag.).

**FIGURE 12:**
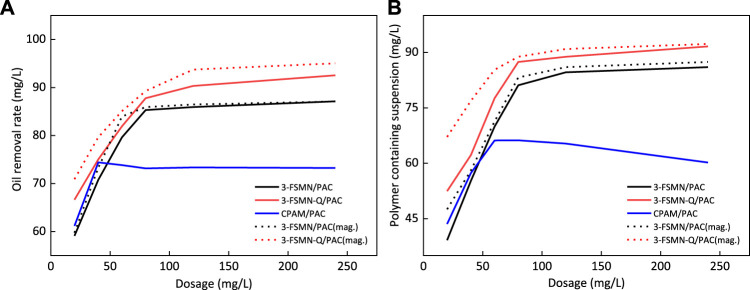
Comparison test of gravity sedimentation and magnetic sedimentation for oil removal rate **(A)** and polymer containing suspended solids removal rate **(B)**.

The solid line in Figure 12 shows the removal rate of oilfield sewage treated with hyperbranched treatment agent combined with polyaluminum chloride PAC under the condition of gravity sedimentation; the dotted line shows the removal rate of magnetic sedimentation of the corresponding treatment agent system. Figure 12A shows the oil removal rate, and Figure 12B shows the removal rate of suspended solids. Obviously, the effect of the magnetic field group of 3-FSMN/PAC and 3-FSMN-Q/PAC is better than that of the natural sedimentation group within 5-min sedimentation time, especially at a low dosage. The quaternized cationic hyperbranched molecule 3-FSMN-Q/PAC can achieve 95% oil removal efficiency and 92.2% suspended solids removal efficiency under magnetic field conditions, and 93.8% oil removal rate and 90% suspended solids removal rate can be achieved with a dosage of 120 mg/L. When the dosage of 3-FSMN/PAC is low, the effect of magnetic sedimentation is better than that of gravity sedimentation. With the increase in dosage, the oil removal capacity tends to be close, which can reach an 87.1% oil removal rate and an 87.3% suspended solids removal rate, and the lower dosage of 120 mg/L can reach an 86.3% oil removal rate and an 86.0% suspended solids removal rate. There is no significant difference between the treatment effect of the cationic polyacrylamide and polyaluminum chloride (CPAM/PAC) system in a magnetic field and gravity sedimentation. The treatment effect of polymer containing wastewater in ASP flooding is not ideal, with the highest oil removal rate of 74.3% and the maximum suspended solids removal rate of 66.1%. With further increase in dosage, the viscosity effect increases and the treatment effect decreases obviously. At a lower dosage, the treatment performance is slightly stronger than that of unmodified 3-FSMN/PAC because the density of 3-FSMN material containing a ferromagnetic core is higher than that of CPAM, resulting in very few reaction active points at a low dosage.

It can be seen that for polymer containing wastewater from ASP flooding, the sewage treatment capacity of 3-FSMN/PAC and 3-FSMN-Q/PAC at appropriate dosage is much better than that of ordinary treatment agent (CPAM/PAC), and the experimental phenomenon is in line with the experimental expectation.

## Conclusion

In this study, nano-Fe_3_O_4_ coated with APTES-modified TEOS was successfully used as a ferromagnetic core. A magnetic hyperbranched polyamide amine was synthesized by Michael addition and ester amidation iterative branching reaction.

The optimum reaction conditions of the first-generation branched product 1-FSMN are a reaction time of 18 h, a reaction temperature of 30°C, a mass ratio of ethylenediamine to FOSN-M of 1:8, the amount of dispersant methanol of 30%, and the yield of 93.7%.

The optimum reaction conditions for the second-generation branched product 2-FSMN were reaction time 24 h, reaction temperature 35°C, methanol solvent dosage 20%, and the ratio of 1-FSMN-M branched molecule to ethylenediamine material 1:15, and the yield was 91.6%. The third- and fourth-generation hyperbranched polymers were prepared under these conditions.

The suspended solids in the experimental sewage are 143 mg/L, the oil content is 921.09 mg/L, the turbidity is 135 NTU, and the zeta potential is −47 MV. The effect of the conventional treatment agent CPAM/PAC compound group on the treatment of polymer containing wastewater in ASP flooding is not ideal. The highest oil removal rate is 74.3% at the dose of 40.3 mg/L. When the dosage is 60.5 mg/l, the maximum removal rate of suspended solids is 66.1%, and then increasing the dosage will not improve the treatment effect.

The third-generation hyperbranched polymer 3-FSMN and its quaternary ammonium salt 3-FSMN-Q perform best in the appropriate dosage range, with the highest oil removal rate of 97%, suspended solids removal rate of 90.3%, turbidity reduction rate of 86.6%, and zeta potential reduction rate of 88%.

For 3-FSMN and its quaternary ammonium salt, the gravity/magnetic PAC compound treatment experiment was carried out. In the settlement time of only 5 min, 3-FSMN/PAC and 3-FSMN-Q/PAC can achieve the maximum oil removal rate of 87.1% and suspended solids removal rate of 87.3% for polymer containing wastewater from ASP flooding, and 86.3 and 86.0% for 120 mg/L (mass ratio FSMN:PAC = 1:1). The treatment capacity is much better than that of ordinary treatment agent (CPAM/PAC).

## Data Availability

The original contributions presented in the study are included in the article/Supplementary Material, further inquiries can be directed to the corresponding author.

## Abbreviations

CPAM, cationic polyacrylamide; Fe_3_O_4_@SiO_2_, spherical products formed by SiO2 deposition on the surface of magnetic nano-Fe_3_O_4_ particles; FOSN, amine-terminated intermediates formed by surface modification of Fe_3_O_4_@SiO_2_; FOSN-M, products of FOSN and methyl acrylate; 1-FSMN, first-generation branched molecules; n-FSMN (n = 1,2,3 … ), Nth-generation hyper/branched molecules; HBP, hyperbranched polymer; PAC, polyaluminum chloride.
